# Thermochemical Laser-Induced Periodic Surface Structures Formation by Femtosecond Laser on Hf Thin Films in Air and Vacuum

**DOI:** 10.3390/ma14216714

**Published:** 2021-11-08

**Authors:** Dmitrij A. Belousov, Kirill A. Bronnikov, Konstantin A. Okotrub, Sergey L. Mikerin, Victor P. Korolkov, Vadim S. Terentyev, Alexander V. Dostovalov

**Affiliations:** Institute of Automation and Electrometry of the SB RAS, Novosibirsk State University, Academician Koptyug Ave. 1, Novosibirsk 630090, Russia; bronnikovkirill@gmail.com (K.A.B.); okotrub@iae.nsk.su (K.A.O.); mikerin@iae.nsk.su (S.L.M.); victork@iae.nsk.su (V.P.K.); terentyev@iae.nsk.su (V.S.T.)

**Keywords:** laser-induced periodic surface structures, hafnium films, digital image processing, microscopy, laser materials processing

## Abstract

Thermochemical laser-induced periodic surface structures (TLIPSS) are a relatively new type of periodic structures formed in the focal area of linear polarized laser radiation by the thermally stimulated reaction of oxidation. The high regularity of the structures and the possibility of forming high-ordered structures over a large area open up possibilities for the practical application for changing the optical and physical properties of materials surface. Since the mechanism of formation of these structures is based on a chemical oxidation reaction, an intriguing question involves the influence of air pressure on the quality of structure formation. This paper presents the results on the TLIPSS formation on a thin hafnium film with fs IR laser radiation at various ambient air pressures from 4 Torr to 760 Torr. Despite the decrease in the oxygen content in the ambient environment by two orders of magnitude, the formation of high-ordered TLIPSS (dispersion in the LIPSS orientation angle δθ < 5°) with a period of ≈700 nm occurs within a wide range of parameters variation (laser power, scanning speed). This behavior of TLIPSS formation is in agreement with experimental data obtained earlier on the study of the kinetics of high-temperature oxidation of hafnium at various oxygen pressures.

## 1. Introduction

Laser-induced periodic surface structures (LIPSS) were discovered for the first time by Birnbaum [1] five decades ago and since then have attracted great interest for a theoretical explanation of the mechanism of structure formation and because of their potential for practical application. LIPSS with the structures period comparable to the laser radiation wavelength and orientation perpendicular to laser polarization are generated within the focusing area of laser radiation. The formation of these structures was obtained on almost any material, including metals [2,3], semiconductors [4,5,6], and dielectrics [7,8]. Although to date a comprehensive theory of LIPSS formation is not available, some theoretical approaches have been proposed for describing the mechanisms of structures formation, based on hydrodynamic processes and models based on the interference of the incident laser beam with an electromagnetic wave scattered at the rough surface [9]. Various practical applications of these structures have been demonstrated to modify optical or physical properties of surfaces (for example, to alter tribological properties or wettability of the original surface). In this regard, LIPSS have great application prospects in various fields such as the creation of hydrophobic coatings [10], biomedicine [11], optical absorptance enhancement of metals surfaces [12], decreasing the friction coefficient of materials [13], metal coloration [14] and fabrication of diffraction holograms [15].

The LIPSS formation mechanism based on an ablation process is considered in a lot of articles, where the periodic structures are formed below the initial surface level at maxima of the laser intensity distribution and have orientation perpendicular to the laser polarization direction. However, in addition to the ablation process, the periodic structures can be formed by means of other mechanisms: photoreduction processes of graphene oxide film [16], thermal—driven modification processes at maxima of the periodic laser intensity distribution, for instance, a modification from a crystalline to an amorphous phase resulting in periodic structures of alternating amorphous-crystalline fringes in case of semiconductors [17], melting in case of polymers [18] or oxidation process in case of metals and semiconductors [19,20]. In the last case, LIPSS formation is based on thermo-stimulated reaction of oxidation that leads to periodic structures formation consisting of alternating areas of the oxidized ridges and unmodified metal with the orientation parallel to the laser polarization direction. This type of thermochemical LIPSS (TLIPSS) is characterized by a high degree of ordering and has great potential for practical applications, especially for cost-effective micro-nanostructuring of surfaces in comparison with expensive lithography-based techniques.

Since the formation of TLIPSS is based on the thermally stimulated process of metal oxidation, one intriguing question is how strongly the concentration of oxygen molecules in the environment affects the process of formation and quality of TLIPSS. The answer can be realized by studying the formation of TLIPSSs in a vacuum in comparison with the results obtained in the air. In the case of ablative structures the decrease in high spatial frequency LIPSS periodicity with increasing ambient air pressure was shown and was explained in terms of the pressure influence on the Marangoni flow of the molten liquid [21]. Also, it was shown that the regularity of ablative structures formed on a thin Cr film that increases with a decrease in ambient air pressure, associated with a weakening of the oxidation process, which disrupted the ordering of ablative structures formed in the atmosphere [22]. Additionally, the structure transition from thermochemical to ablative LIPSS is demonstrated on 50 nm thick Cr film deposited on Si substrate via varying the residual air pressure in a vacuum chamber [23]. Thermochemical LIPSS are predominantly formed at atmospheric pressure, but with a 50-fold decrease in pressure, the appearance of ablative structures was observed alongside thermochemical LIPSS, which completely dominate with a further decrease in oxygen pressure to a high vacuum. These structures formation transition is also explained in [22] by a weakening of the oxidation process at low air pressure and the consequences of competitive excitation of the transverse-electric scattered surface wave and transverse-magnetic hybrid plasmon wave depending on the oxidation degree.

Hafnium is an interesting and technologically important material due to its relatively high melting point of 2506 K (in fact, it has the highest melting point of all metals in the Ti subgroup), which makes Hf useful in high-temperature applications. Its stable oxide, HfO_2_, also has a high melting point of 3030 K and is widely adopted in electronics as an insulator, having a several times higher dielectric constant than that of silicon dioxide. It is also applied as a material for anti-reflective coatings. Considering the technological importance of HfO_2_, the development of a highly productive method of fabricating large-scale HfO_2_ structures with near-wavelength periodicity is a challenge. Moreover, creating periodic patterns is interesting for plasmonic applications. For example, it is a straightforward process to cover regular TLIPSS with some low-loss metal, such as silver or gold, to excite surface plasmons in order to significantly increase the photoluminescence intensity in the label-free detection and imaging of biomolecules [24].

This paper presents the results of the study on the formation of TLIPSS with a quantitative assessment of the parameters of ordering, defectiveness and productivity of the creation of periodic structures formed on thin hafnium films when exposed to fs laser radiation at different concentrations of oxygen molecules in ambient atmosphere.

## 2. Materials and Methods

**Figure 1:**
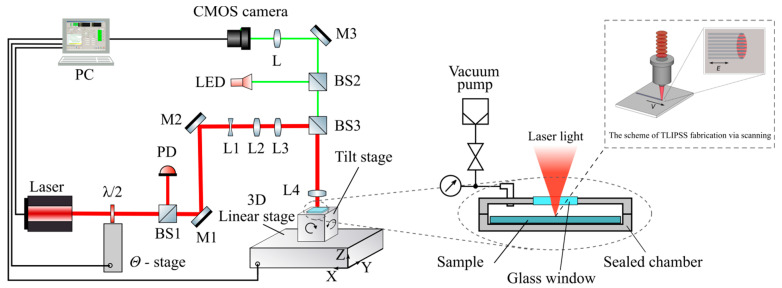
Experimental setup for TLIPSS formation in air and a low vacuum.

Metal films of hafnium, 15 nm thick, were deposited on borosilicate glass substrates by magnetron sputtering. The surface roughness of Ra ≈ 0.5 nm was measured by atomic-force microscopy. The scheme of the experimental setup is shown in Figure 1. Femtosecond radiation with wavelength of 1026 nm, pulse repetition rate of 200 kHz, pulse duration of 232 fs is used. The laser radiation after passing through the λ/2 plate, the angular position of which allows controlling the direction of the polarization of the incident light, goes through the cylindrical concave lens (L1) with *f*_L1_ = ‒1 m and convex lenses with *f*_L2_ = 28 mm (L2), *f*_L3_ = 14 mm (L3). The lens L1 converts a Gaussian symmetrical beam into an astigmatic one, and the lenses L2 and L3 determine the beam size at the entrance of the focusing lens. Finally, the radiation is focused on the surface of the metal film using the convex lens *f*_L4_ = 50 mm (L4). The astigmatically focused Gaussian beam had an elliptical focal spot with an aspect ratio of 1:10 and a size along the major axis ≈ 150 μm to increase the productivity of the TLIPSS formation. The laser power (P) and processing speed (V) was varied from 200 to 300 mW and from 100 to 2000 μm/s, respectively.

The TLIPSS formation in a vacuum was carried out on the sample in a gas chamber, from which air was evacuated using a vacuum pump (residual pressure—4 Torr). The fs laser radiation was focused on the sample through a transparent fused silica window 3 mm thick.

The estimation of TLIPSS parameters was carried out by analyzing images obtained with a scanning electron microscope (SEM) Hitachi TM3000. Using the technique described in detail below, and also in the Appendix A, the following TLIPSS parameters were determined: TLIPSS formation productivity P_sw_ [μm^2^/s], relative area of defects D [%], regularity of structures (dispersion in the LIPSS orientation angle, DLOA) δθ [deg.] and Ξ [a.u.], regularity of structures in the area where TLIPSS are formed effectively (parameters δθ_eff.area_ [deg.] and Ξ_eff.area_ [a.u.]). Raman spectra were measured in backscattering geometry using a solid-state single-mode laser at 532.1 nm. Laser beam with a power of 0.5 mW was focused into a region ~ 1 µm in size. The spectra were averaged over an area of 160 × 160 μm. Baseline was subtracted using linear function and substrate contribution was also subtracted.

To quantitatively evaluate the TLIPSS formation productivity and defectiveness, pixels $Pix_{\sum }$ on the SEM image, which refer to the TLIPSS on the processed SEM image, and pixels $Pix_{D}$, which characterize defective areas, were determined (see Appendix A for more details). The relative area of defects of the TLIPSS in this case is determined by the following expression:

(1) $$D=\frac{Pix_{D}}{Pix_{\sum }}\times 100\%$$

**Figure 2:**
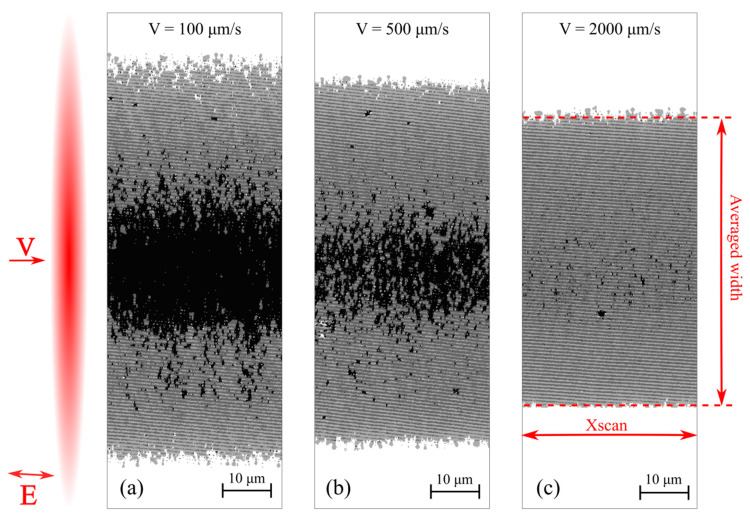
Processed SEM images formed in air at a power of 250 mW and different scanning speeds: 100 μm/s (**a**), 500 μm/s (**b**), 2000 μm/s (**c**). The inset shows schematically the astigmatic beam, directions of scanning and polarization.

The productivity of structures formation is determined as the product of the average width of the structure *W* (Figure 2c), obtained by single-pass scanning of the sample, by the scanning speed *V*:

(2) $$P_{SW}=W\times V=\frac{Pix_{\sum^{\;}}\times Pix_{SY}}{X_{scan}}\times V\;$$

where $Pix_{SY}$—physical size of a pixel in a image in the direction perpendicular to the scanning direction, $X_{scan}$—the length of the registered modified surface (in pixels) on the processed SEM image (Figure 2c).

**Figure 3:**
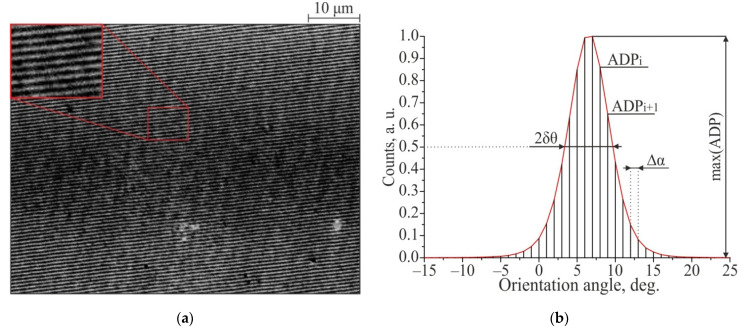
Determination of the regularity of the TLIPSS formed at a laser power of 250 mW and a scanning speed of 500 μm/s: a fragment of the original SEM image (**a**); angular pixels distribution (ADP) graph (∆*α* = 1°) (**b**).

The proposed method for determining the regularity of structures is based on the approach described in [25], in which, using tensor analysis of the processed image, the angular orientation of each pixel in the SEM image is determined, then the angular distribution of pixels is obtained, and dispersion in the LIPSS orientation angle (DLOA) δθ is determined [25]. Subsequent summation of the number of pixels with the angular orientation from a given interval ∆*α* of a selected sampling step in angle, allows us to plot the angular distribution of pixels (ADP). The parameter DLOA δθ is defined as the half-width at half maximum of this graph (value δθ in Figure 3). This parameter is comparable in magnitude with the parameter of angular opening cone of the two-dimensional Fourier transform of the original SEM image, and characterizes the straightness of the TLIPSS. As in [25], the freely available plug-in OrientationJ [26], developed for the open source software ImageJ [27], was used to determine the DLOA parameter.

In addition to the DLOA δθ parameter, to assess the general regularity of structures, we propose to consider the parameter Ξ [a.u.], which characterizes the parallelism of the structure, and which is equal to the area under the normalized graph of the angular distribution of pixels:

(3) Ξ=∆α×∑i=1180∆α (ADP)imax(ADP)

where max(ADP)—maximum of the angular pixels distribution graph (Figure 3). An increase in the area under the normalized ADP graph corresponds to an increase in the number of pixels (in percentage) with a local angular orientation that does not coincide with the main direction of the angular orientation of the TLIPSS. The parameter Ξ varies in the range from ∆*α* to 180, where the value of ∆*α* corresponds to the case of a perfectly parallel structure, and the value of 180 to the case of a circular ring structure, in which all possible local angular orientations are equally presented.

Thus, a quantitative assessment of the regularity of the TLIPSS is based on the analysis of SEM images and determination of the parameters DLOA δθ (the parameter of the structures straightness) and Ξ (the parameter of the structures parallelism) obtained from an angular-pixels distribution graph.

Using, for the ADP graph, only pixels that correspond to the area where TLIPSS are formed effectively, allows researchers to determine the parameters δθ_eff.area_ and Ξ_eff.area_. This allows one to minimize the error of the determination of TLIPSS regularity arising from the defects area of the structures. Therefore, both sets of parameters δθ, Ξ and δθ_eff.area_, Ξ_eff.area_ are used to characterize the TLIPSS regularity.

## 3. Results and Discussion

**Figure 4:**
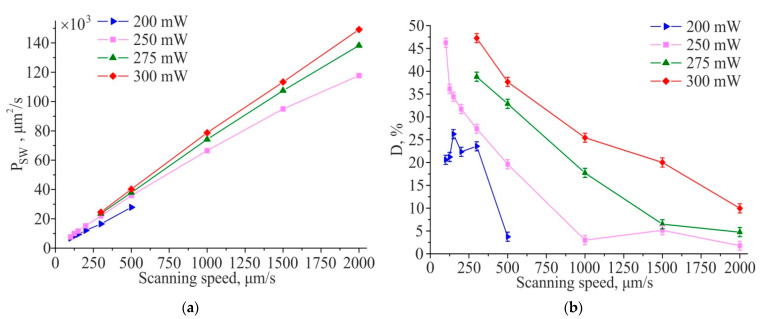
Productivity of TLIPSS formation on a hafnium film in air (**a**) and relative area of defects (**b**) at different scanning speeds and laser powers.

Figure 4a shows the productivity of TLIPSS formation with structures period of ≈700 nm on a hafnium film in air and relative area of defects obtained at different laser power and processing speeds. The results show that in the used ranges of powers and scanning speeds, the productivity increases with an increasing in scanning speed. It is interesting to note that, as shown in Figure 4b, the relative defect area significantly decreases with increasing scanning speed. A large area of defects is concentrated in the central region of the formed TLIPSS at “low” scanning speeds due to overexposure of the material at maximum of Gaussian intensity distribution. In particular, for a scanning speed of 300 μm/s, the value of the relative area of defects D, depending on the power, varies from 24% to 47%. As seen in Figure 2, an increase in the scanning speed leads to a decrease in the exposure dose in the central region of the TLIPSS and the formation of a regular periodic structure in this area, which results in the decrease in relative area of defects. In particular, for a laser power of 250 mW and a scanning speed of 2000 µm/s, the relative area of defects *D* is about 1.5–2%. But the relative defect area D increases up to 4.5% and 10% at the same scanning speed for a beam power of 275 and 300 mW, respectively.

**Figure 5:**
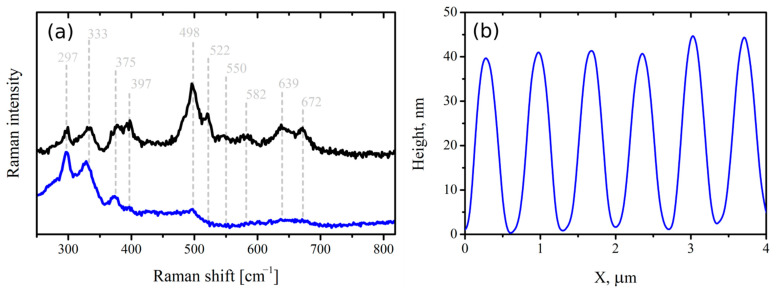
(**a**) Raman spectra of TLIPSS structures. The black line is the spectrum measured from TLIPSS formed on thick (150 nm) coating. The blue line corresponds to TLIPSS formed on 15 nm thick coating at 250 mW and 2000 μm/s. Spectra vertically shifted for clarity. (**b**) AFM profile of TLIPSS formed at 250 mW and 2000 μm/s.

In order to characterize the chemical composition of TLIPSS, Raman spectra were measured (Figure 5a). Two TLIPSS samples formed on Hf film of different thickness were tested. The first structure was formed on 150 nm coating. The Raman spectrum of this structure contains Raman peaks at 397, 498,522, 550, 582, 639, and 672 nm assigned to monoclinic crystalline HfO_2_ [28]. The structures formed on 15 nm film formed at 250 mW and 2000 μm/s also demonstrate faint spectral features at frequencies related to the Raman spectrum of HfO_2_. Low intensity of these peaks is due to the low thickness of Hf coating. In addition, both spectra contain unassigned Raman peaks at 297, 333, and 375 cm^−1^, probably related to modification of the glass substrate material. Raman spectrum of unmodified hafnium coating does not demonstrate any significant spectral features. Thus, the appearance of new Raman lines indicates the thermochemical mechanism of LIPSS formation. The AFM profile of TLIPSS formed at 250 mW and 2000 μm/s was presented in Figure 5b showing the height of oxide ridges of 40 nm, which can be explained by several reasons. Firstly, the Hf oxide occupy more volume (V_HfO2_) than metal (V_Hf_) because the Pilling–Bedworth ratio for HfO_2_/Hf R_PB_ = V_HfO2_/V_Hf_ = 1.62. Moreover, it was previously shown that the metal oxide in TLIPSS ridges is porous, resulting in an increase in volume in comparison with solid oxide [29]. Also, the significant height of oxide ridges in comparison with metal film thickness could be explained by glass substrate melting under the oxide ridges resulting in a rise of the initial glass surface after glass resolidification because of the lower density of melted glass [30].

**Figure 6:**
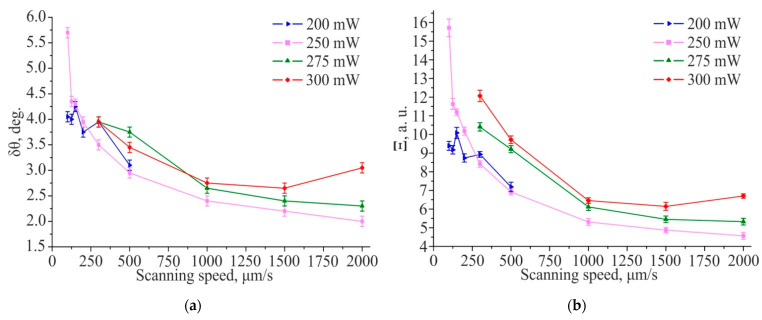

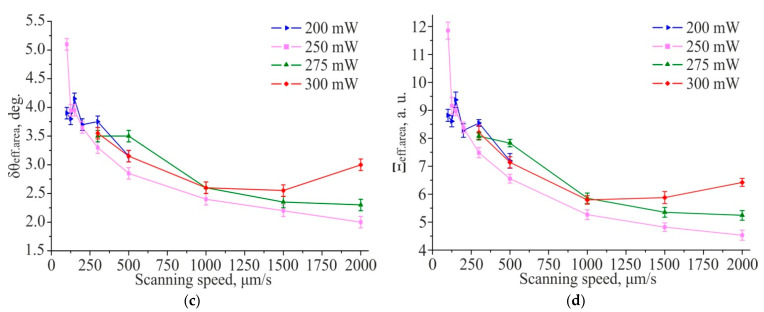
Dependences of the structures straightness δθ (**a**) and the structures parallelism Ξ parameters (**b**) for TLIPSS formed on a hafnium film in air at different scanning speeds and laser powers, corresponding values for δθ_eff.area_ (**c**) and Ξ_eff.area_ (**d**).

As shown in Figure 6, TLIPSS formed on hafnium films in air, within the used ranges of power and scanning speed, have a high degree of ordering (DLOA δθ < 4°). It is interesting to note that with an increase in the scanning speed from 100 μm/s to 2000 μm/s, the regularity of TLIPSS formed on hafnium films improves from DLOA δθ ≈ 5° to δθ ≈ 2°.

Thus, in the studied ranges of laser powers and scanning speeds, all key parameters (relative area of defects and regularity) of TLIPSS improve with an increase in the scanning speed, leading to an increase in productivity of TLIPSS formation. Moreover, in comparison with chromium films, for which the maximum speed of ordered structure formation under the same focusing conditions does not exceed 100 μm/s [31], as in the case of hafnium films, the maximum processing speed (2000 μm/s) for regular TLIPSS formation increases by an order of magnitude. Thus, the results obtained show that hafnium is a promising material for the high-throughput formation of TLIPSS with a high structures regularity. It is likely that the quality of the formed structures can be further improved by using a top-hat beam with a uniform power distribution within the focal spot [32].

**Figure 7:**
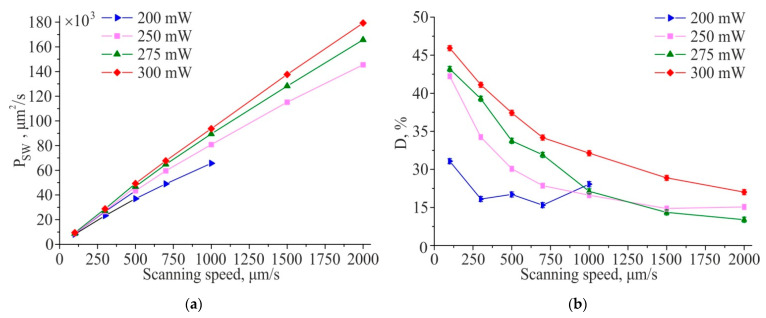
Productivity of TLIPSS formation on a hafnium film in a low vacuum (**a**) and relative area of defects (**b**) at different scanning speeds and laser powers.

Since the formation of TLIPSS requires the presence of oxygen for the oxidation reaction, to determine the influence of the ambient oxygen concentration on TLIPSS formation, experiments were also carried out in a low vacuum (residual air pressure of 4 Torr). Productivity of TLIPSS formation on a hafnium film in a low vacuum and relative area of defects at different scanning speeds and laser powers are shown in Figure 7b.

The minimum relative area of defects D of the formed structures in a low vacuum is ~10% (at laser power of 275 mW and a scanning speed of 2000 μm/s), which is significantly higher in comparison with structures formed in a standard air atmosphere (D = 2%). However, the results obtained indicate the possibility of the formation of oxide structures even with a decrease in the oxygen concentration by several orders of magnitude.

**Figure 8:**
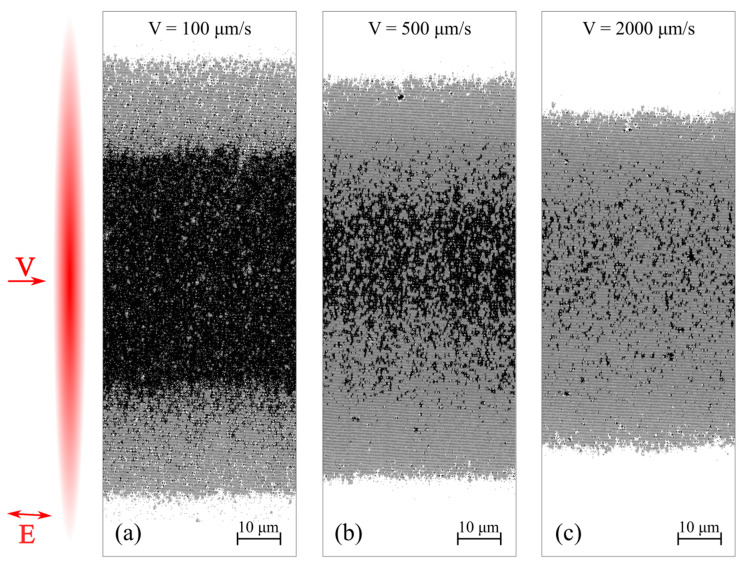
Processed SEM images formed in a low vacuum at a power of 250 mW and different scanning speeds: 100 μm/s (**a**), 500 μm/s (**b**), 2000 μm/s (**c**). The inset shows schematically the astigmatic beam, directions of scanning and polarization.

**Figure 9:**
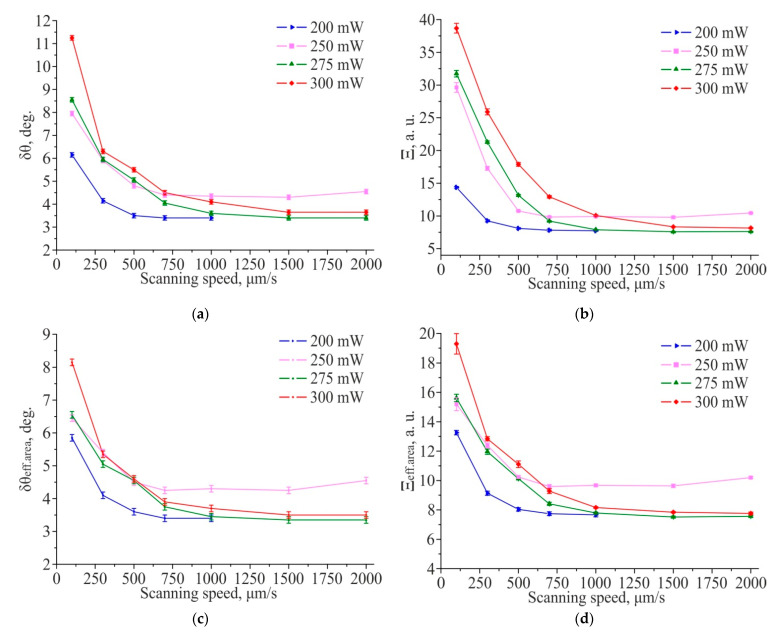
Dependences of the structures’ straightness δθ (**a**) and the structures’ parallelism Ξ parameters (**b**) for TLIPSS formed on a hafnium film in a low vacuum at different scanning speeds and laser powers, corresponding values for δθ_eff.area_ (**c**) and Ξ_eff.area_ (**d**).

As shown in Figure 8, an improvement in the structures’ regularity with an increase in the scanning speed for TLIPSS formed on hafnium films in a low vacuum is observed, as well as for TLIPSS formation in an air atmosphere. However, their regularity is worse compared to the structures formed in an air atmosphere. At laser power of 200 mW, the best regularity (DLOA δθ = 3.5°) was achieved at a scanning speed of 1000 μm/s (Figure 9). For a power of 275 mW at a scanning speed of 1000 μm/s, the parameters of regularity reached a minimum value of 3.5° and did not change significantly with a further increase in the speed. Consequently, for a given power, the scanning speed of 2000 μm/s is close to optimal for TLIPSS formation in a low vacuum. For a power of 300 mW, the DLOA δθ parameters of the structures reached 3.6° at a scanning speed of 1500 μm/s.

Thus, despite the decrease in the oxygen concentration in the ambient environment by two orders of magnitude, the formation of ordered TLIPSS occurs within a wide range of variation in parameters (laser power, scanning speed). This behavior of TLIPSS formation is in agreement with experimental data on the study of the kinetics of high-temperature oxidation of hafnium at various oxygen pressures [33], Where it was shown that in the temperature range of 1000–1200 °C, when the pressure changes from 0.1 to 760 Torr, the kinetics of the oxidation process does not depend on the oxygen pressure. At lower temperatures (650–850 °C), oxidation kinetics is also weakly dependent on oxygen pressure [34]. Moreover, in contrast to [22], the change in pressure by a factor of 200 did not significantly affect the type of structures formed, which can be explained by the more efficient oxidation of metals of the titanium subgroup in comparison with chromium, because of larger parabolic oxidation rate constants of Hf in comparison with Cr [35].

**Figure 10:**
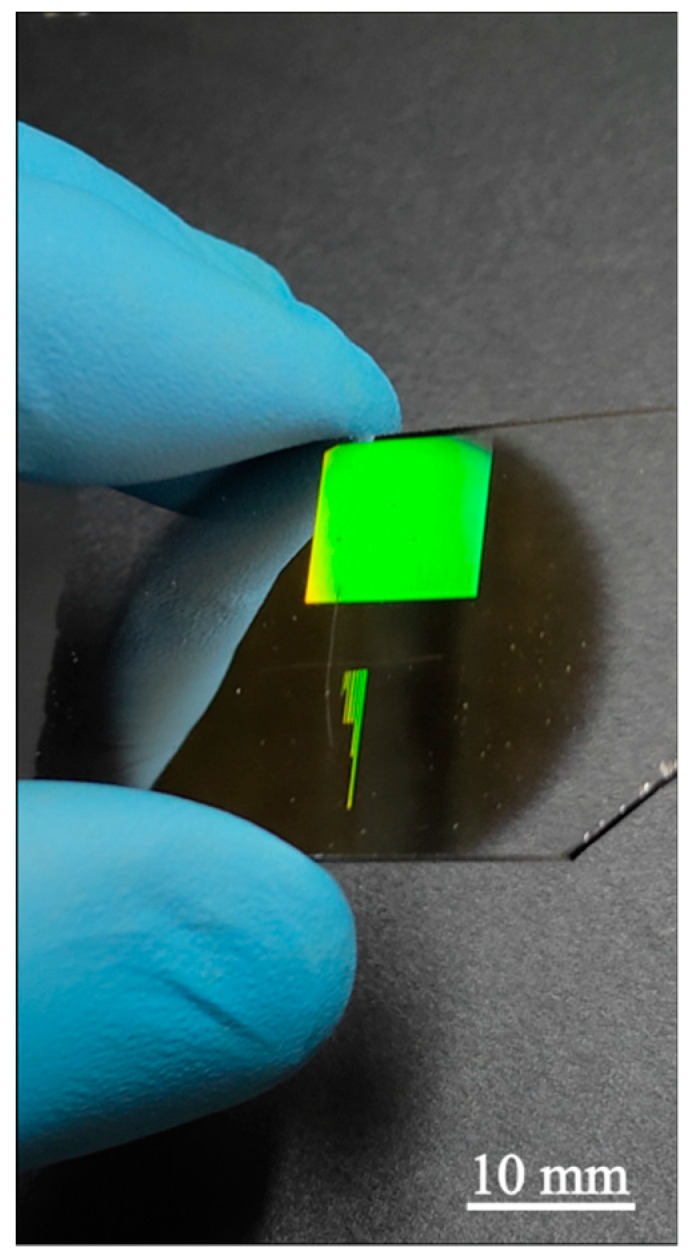
Structural colors on a Hf thin film of 10 × 10 mm^2^ obtained from white light illumination. The corresponding LIPSS pattern was obtained in air at 2000 um/s and 250 mW.

To demonstrate the possibility of high ordered structures formation over a large area, a structure of 10 × 10 mm^2^ in air at scanning speed of 2000 um/s and laser power of 250 mW was formed. Structural colors on a Hf thin film obtained from white light illumination is presented in Figure 10, where an ordered structure is formed over the entire area.

## 4. Conclusions

As a result, the formation of thermochemical laser-induced periodic surface structures with fs IR laser radiation on a thin (15 nm) hafnium film was investigated at various ambient air pressures from 4 Torr to 760 Torr. In the case of a pressure of 760 Torr, the formation of highly ordered TLIPSS (dispersion in the LIPSS orientation angle δθ in range of from 2 to 5°) with a period of ≈700 nm in a wide range of variation of the scanning speed (100–2000 μm/s) and radiation power (200–300 mW) is shown. It was found that with an increase in the scanning speed, the regularity parameters of the TLIPSS formed on hafnium films improve, so at laser power of 250 mW, an increase in the scanning speed from 100 μm/s to 2000 μm/s leads to a decrease in DLOA δθ ≈ 5° to δθ ≈ 2° and to a decrease in relative area of defects D from 47 to 2%. Whereas the productivity of the structures formation in this case increases by more than an order of magnitude from 10 to 110 μm^2^/s.

With a decrease in ambient air pressure to 4 Torr, the formation of the TLIPSS with a period of ≈700 nm is also demonstrated within the same ranges mentioned above of processing parameters. As in case of the TLIPSS formation in air at 760 Torr, an increase in the structure regularity with an increase in the scanning speed for TLIPSS formation in a low vacuum is observed. However, in this case, the structures regularity of DLOA δθ = 3.5 ° is worse than in the case of TLIPSS formation at 760 Torr. Thus, despite the decrease in the oxygen content in the ambient environment by 2 orders of magnitude, the formation of ordered TLIPSS occurs within a wide range of parameters variation (laser power, scanning speed). This behavior of TLIPSS formation is in agreement with experimental data obtained earlier on the study of the kinetics of high-temperature oxidation of hafnium at various oxygen pressures [33,34].

Thus, the obtained results indicate that hafnium is a promising material for the high-throughput formation of high-ordered periodic structures for different possible applications in biomedicine [36], photovoltaic [37], improving tribology properties [38], metal coloration and fabrication of diffraction holograms [15].

## Figures and Tables

**Figure A1:**
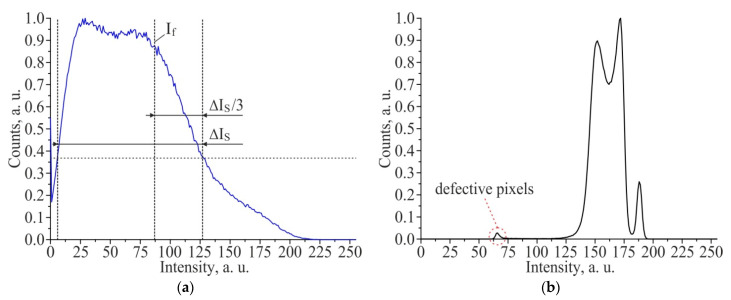
Analysis of the graph of the distribution of pixel intensity on the SEM image of TLIPSS: highlighting the light and dark areas of the investigated periodic structure (**a**); an example of the selection of defective pixels by analyzing the intensity distribution graph (**b**).

**Figure A2:**
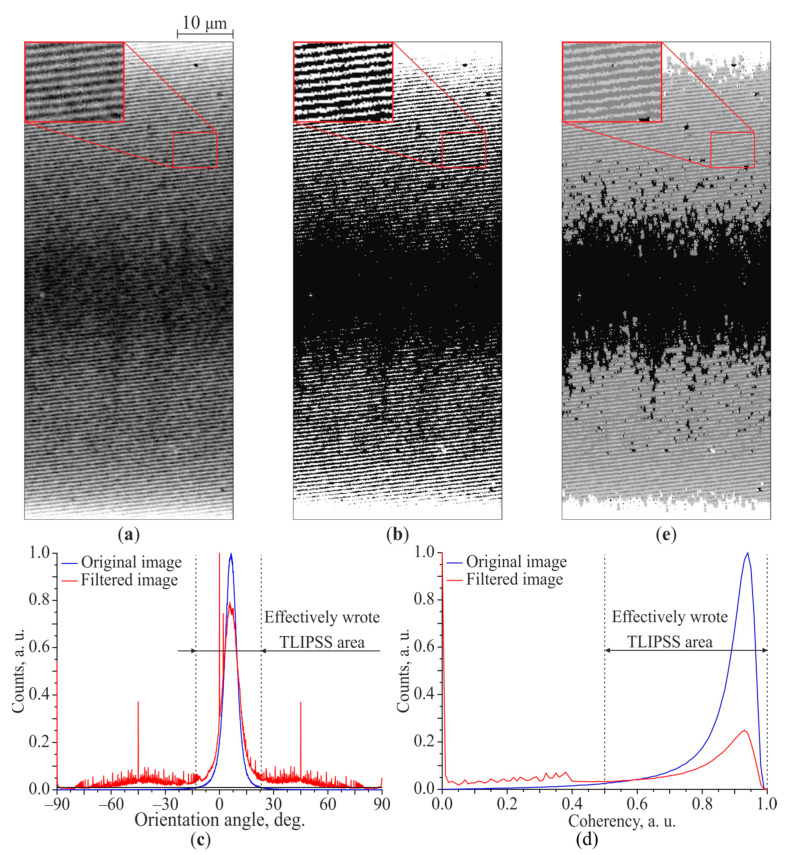
Results of analyzing the SEM images of TLIPSS: the fragment of the original image (writing parameters: P = 250 mW, V = 500 μm/s) (**a**); the contrast image after filtering by intensity (**b**); graphs of angular distribution (**c**) and coherency (**d**) of pixels; the image after determining the areas of defects of TLIPSS and areas not modified by laser beam (**e**).

**Figure A3:**
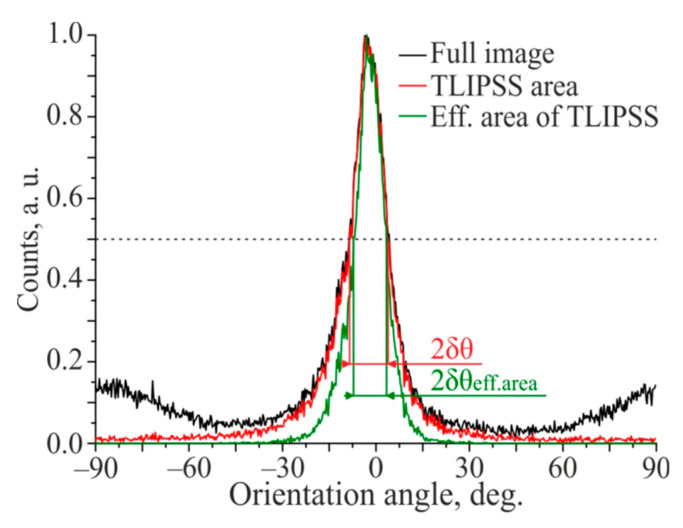
The ADP graphs for different areas of the TLIPSS image, formed on a hafnium film by the immobile astigmatic Gaussian beam at a power of 300 mW.

## Data Availability

The data presented in this study are available on request from the corresponding author.

## Appendix A

To quantitatively evaluate the writing productivity of TLIPSS and area of defects of the formed structures on the SEM image, it is necessary to determine the pixels that characterize the regions not modified by laser beam radiation and the pixels that characterize the defective areas on the formed periodic structure. For these purposes, in this article, an approach based on the use of data on the angular orientation of pixels and their angular coherence is applied. The coherence value is varied between 0 and 1, with 1 indicating highly oriented structures and 0 indicating isotropic areas [39]. To obtain this information, as well as to determine the oxide structures regularity, the data obtained during the analysis of the SEM image in the ImageJ program (OrientationJ plugin) are used. This makes this approach highly convenient, since it allows us to use the same tool to determine all the parameters of the TLIPSS formation quality that are of interest to us (productivity and relative area of defects of the writing, as well as the regularity of the formed TLIPSS).

According to the proposed method, for a start, by analyzing the intensity distribution on the processed SEM image, the intensity range is determined, which characterizes the main part of the investigated structure (in order to create a contrast image of TLIPSS). For this, a graph of the distribution of pixel intensities (Figure A1a) on the processed SEM image is built (the given graph corresponds to the processing SEM image, a fragment of which is shown in Figure A2a). On the resulting graph, at the «1/e» level, the range of pixel intensities related to the main investigated structure ∆I_S_ is determined. Then the areas that are characterized by lighter pixels are defined. In the investigated SEM images, lighter pixels correspond to metal area on the metal/oxide structure of TLIPSS and to areas of the metal film around the structure not modified by the laser beam. Since during image processing in the ImageJ program, the duty cycle of structures does not affect the determination of the angular orientation of pixels, it is not necessary to accurately determine the border of oxide and metal areas (dark and light areas on the SEM image) of TLIPSS. In this regard, in order to highlight the light and dark areas of the investigated periodic structure on the image, the filter value is set by intensity I_f_, spaced from the right border of the ∆I_S_ interval by ∆I_S_/3, as shown in (Figure A1a). To create an image with highlighted light areas (contrast image), all pixels whose intensity is greater than or equal to the I_f_ value are assigned a value of 255, and those pixels whose intensity is less than the I_f_ value are assigned a value of 10. If the processed SEM image contains pixels that can be identified as defective at the stage of analyzing the intensity distribution (Figure A1b), then they are assigned a value equal to 0. Thus, an image is formed in which metal area and areas not modified by the laser writing beam (white areas in Figure A2b) have a pronounced contrast in comparison with the regions of the formed oxide on the investigated structure (black areas in Figure A2b). This makes it possible to distinguish a uniformly formed structure on the investigated SEM image (excluding low-contrast areas).

The Appendix A is an optional section that can contain details and data supplemental to the main text—for example, explanations of experimental details that would disrupt the flow of the main text but nonetheless remain crucial to understanding and reproducing the research shown; figures of replicates for experiments of which representative data is shown in the main text can be added here if brief, or as Supplementary data. Mathematical proofs of results not central to the paper can be added as an Appendix A.

Furthermore, in the OrientationJ plugin, the original SEM image (Figure A2a) and the resulting contrast image (Figure A2b) are processed. To determine the pixels, which characterize the areas not modified by laser beam, and areas of defects of TLIPSS, maps of the angular orientation of pixels and their angular coherence, obtained as a result of the analysis SEM images, are used. Based on the obtained maps, the corresponding graphs are built: ADP graphs (Figure A2c), and graphs of coherence distribution of pixels (Figure A2d). On the obtained ADP graph for the original SEM image, boundaries of the “dome” are determined, which characterizes the main range of angular orientations of the structures (Figure A2c). In this work, to determine of this «dome» boundary, a filter is used, set at a level of 1% of the difference between the maximum and minimum values on the obtained ADP graph (Figure A2c). If the angular orientation of a pixel with coordinates (x_m,n_,y_m,n_) falls into the main angular range of the investigated structure in both images (Figure A2c), and at the same time its angular coherence in both images > 0.5 (that is, a pixel characterizes an oriented structure, as shown in Figure A2d), then this pixel characterizes the effectively formed TLIPSS area. If this condition is not met, then this pixel characterizes the area of defects of the investigated structure, if it belongs to the area of the formed oxide (black pixels in the contrast image, as shown in Figure A2b), or the area not modified by laser beam, if it belongs to the area of the metal (white pixels in the contrast image, as shown in Figure A2b). The result of processing the SEM image of the TLIPSS by the described method is shown in Figure A2e. In this image, white pixels characterize areas not modified by laser beam, and black pixels, which characterize areas of defects on the investigated structure, and TLIPSS in an effectively formed area are represented by shades of gray.

Determining the pixels that characterize the effectively formed TLIPSS area is also important for quantifying assessment of oxide ripples regularity. In the general case, when all pixels characterizing the investigated structure are used to construct the ADP graph, the TLIPSS defect areas can make a certain contribution to the determination of the oxide ripples regularity parameters (DLOA δθ and Ξ). Plotting the ADP graph using only pixels that characterize the effectively formed TLIPSS area (Figure A3) allows for the determination of the corresponding parameters DLOA δθ_eff.area_ and Ξ_eff.area_. This makes it possible to minimize the error introduced by the defective areas of the TLIPSS in the determination of the oxide ripples regularity of an effectively formed structure.

The analysis of SEM images in the ImageJ program using the OrientationJ plugin was performed in the Orientation Distribution module with the Gaussian Gradient structural tensor. No Gaussian smearing was applied. The ADP graphs in this work were constructed by analyzing the maps of the angular orientation of pixels on the investigated images. To do this, after processing the SEM image in the OrientationJ plugin, the “Orientation” tab is selected and the resulting map of the angular orientation of pixels is saved. The ADP graph is constructed by summing the number of pixels, the angular orientation of which falls within a given interval with a selected sampling step for the orientation angle ∆*α*. The sampling step ∆*α* was chosen in the range from 1° to 0.1°, starting with the larger value. The criterion for choosing the interval ∆*α* is the condition of the convergence of the results when determining the value δθ (the value of the DLOA parameter). The DLOA parameter for the selected ∆*α* value lies in the range δθ(∆*α*) ± ∆*α*. With a decrease in ∆*α*, the value of the DLOA parameter should not go beyond the range obtained with a larger value of ∆*α*. If this condition is not met, then a further decrease in the sampling step in angle ∆*α* is not performed.
